# Case report of a giant kidney stone removed by transperitoneal laparoscopic pyelolithotomy

**DOI:** 10.1186/s12894-025-01959-5

**Published:** 2025-10-29

**Authors:** Ruslan N. Trushkin, Teymur K. Isaev, Pavel E. Medvedev, Petr P. Teihrib, Mariana A. Lysenko, Ilya V. Dmitriev, Aslan G. Balkarov, Tuong Lan Ho

**Affiliations:** 1 Department of Urology, Moscow City Clinical Hospital № 52 of Moscow Healthcare Department, Moscow, Russian Federation; 2 Department of Kidney and Pancreas transplantation, Sklifosovsky Research Institute for Emergency Medicine of Moscow Healthcare Department, Moscow, Russian Federation; 3 Chair of Transplantology and Artificial Organs, Department of Continuous Medical Education, Pirogov Russian National Research Medical University, Ministry of Health of the Russian Federation, Moscow, Russian Federation; 4 Department of Urology and Operative Nephrology with a course of oncourology. Peoples’, Friendship University of Russia named after Patrice Lumumba, Moscow, Russian Federation

**Keywords:** Giant kidney stone, Staghorn stone, Laparoscopic pyelolithotomy

## Abstract

**Introduction:**

In cases of long-standing giant kidney stones resulting in irreversible loss of renal function, frequent urinary tract infections and a chronic flank pain that cannot be managed by other treatment options nephrectomy is considered the only effective treatment option. The largest KS documented in the literature weighed 2260 g and was treated by nephrectomy; while the largest stone removed without nephrectomy weighed 1350 g. There are no documented cases of KS over 200 g being removed by laparoscopic surgery. The preserved excretory function supported proceeding with pyelolithotomy rather than nephrectomy.

**Objective:**

We hereby present a case of a giant KS in a functioning kidney of the female patient. She had previously (> 15 years ago) undergone an open left nephrolithotomy which made the procedure more challenging. She underwent successful transperitoneal laparoscopic pyelolithotomy.

**Materials and methods:**

The patient, aged 55, underwent an kidney-preserving transperitoneal laparoscopic left-sided pyelolithotomy due to the presence of a large staghorn stone in a functioning kidney. Because kidney function was salvageable, nephrectomy was not considered.

**Results:**

A successful kidney-preserving surgery was performed, without the occurrence of any surgical complications. The postoperative period was characterized by a brief duration of hospital stay.

**Conclusion:**

Laparoscopic pyelolithotomy is a safe and effective surgical technique for the removal of large and complex KS. The minimally invasive approach ensures a rapid recovery process, minimal blood loss, reduced postoperative pain, and improved cosmetic results. However, it should be noted that the method requires highly skilled surgeons and advanced laparoscopic instruments were used (30° laparoscope, trocars, atraumatic graspers, maryland dissectors, monopolar/ultrasonic energy, and barbed suture for pyelotomy closure).

## Introduction

Staghorn stones, also referred to as coral stones, account for about 4% of kidney stones (KS) [1]. The presentation of KS is typically accompanied by symptoms such as flank pain, fever, or haematuria. However, in the absence of significant obstruction, these stones may undergo substantial growth, often without any overt symptomatic manifestations. KSs that exceed 100 g in size are extremely rare. In cases of long-standing giant KS, there is an irreversible loss of kidney function, as well as frequent urinary tract infections and a chronic flank pain that cannot be managed by other treatment options. In such cases nephrectomy is considered the only effective treatment option. The largest documented KS, weighing 2260 g, was treated by nephrectomy [2]. The reports on large KS treatment without nephrectomy are limited, with the largest documented case, weighing 1350 g, reported in a patient in Turkey in 2005 [3]. There are no documented cases of KS larger than 200 g being removed by laparoscopic surgery.

## Case report

**Fig. 1:**
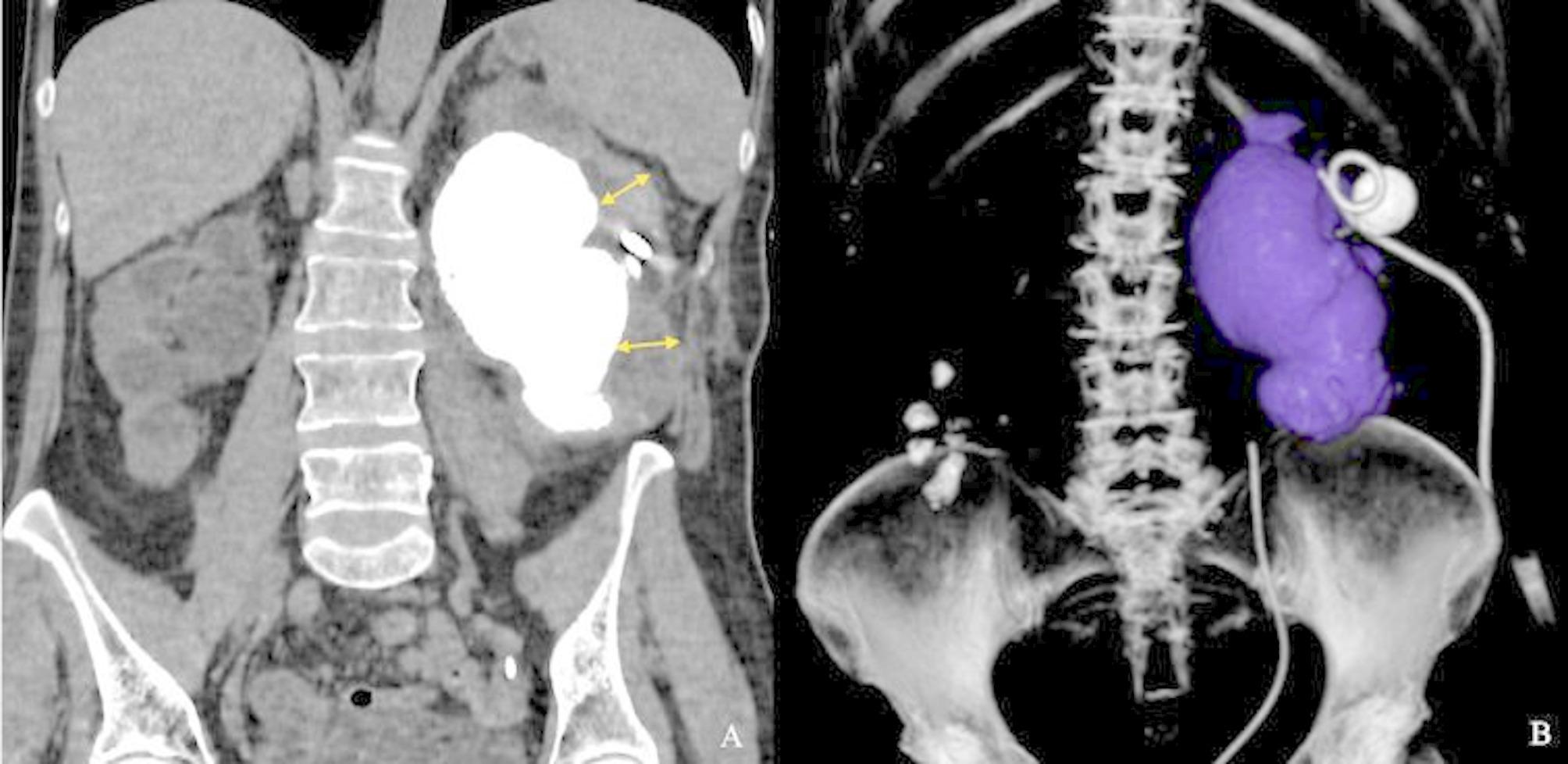
Сontrast-enhanced сomputed tomography. 1 A - parenchymal thickness indicated by the yellow arrows, giant staghorn stone indicated in white; 1B – giant staghorn stone indicated in blue, additional kidney stone indicated in white

A 55-year-old female patient presented with dull aching pain in the left flank. A review of the patient’s medical history revealed that the patient had undergone left pyeloplasty for ureteropelvic junction obstruction in 1989. Subsequently, in 2002, the patient underwent an open left nephrolithotomy. These surgeries were performed at a different hospital and the patient has lost the medical records relating to these hospitalizations. In 2004, the patient was diagnosed with bilateral staghorn stones. Despite being prescribed percutaneous nephrolithotomy, the patient declined surgical intervention. Due to the lack of medical records, it is difficult to assess kidney function and the size of KS before actual hospitalization. In 2024, the patient exhibited an exacerbation of symptoms, including bilateral flank pain, and underwent bilateral nephrostomy placement followed by right percutaneous nephrolithotomy. In September 2024, the patient presented to our hospital for treatment of left KS. Сontrast-enhanced сomputed tomography (CT) revealed multiple small stones in the right renal with a density ranging from 700 to 1000 Hounsfield units (HU). The dimensions of the left kidney were 173 × 75 × 65 mm, and the kidney parenchymal thickness ranged from 9 to 14 mm (Fig. 1A). A large left KS measuring approximately 136 × 80 × 68 mm with a density of 887 HU was identified (Fig. 1B). In addition to the giant staghorn stone, there was another calyceal stone in the.

left kidney (25х22х20 mm). Complete stone removal should be attempted; if residual calyceal fragments remain, a second-stage PCNL can be performed for complete clearance.

The patient’s urine output through the left nephrostomy tube was approximately 500 ml per 24 h and estimated glomerular filtration rate (eGFR) was 23 ml/min. Left antegrade urography revealed no evidence of ureteral stenosis. Given the relatively preserved excretory function of the left renal, a nephron-preserving approach was selected for the stone removal. Taking into account the preserved kidney function and the risks associated with impaired urinary passage due to the large stone, the operation was reasonable. Because kidney function was salvageable, nephrectomy was not considered.

**Fig. 2:**
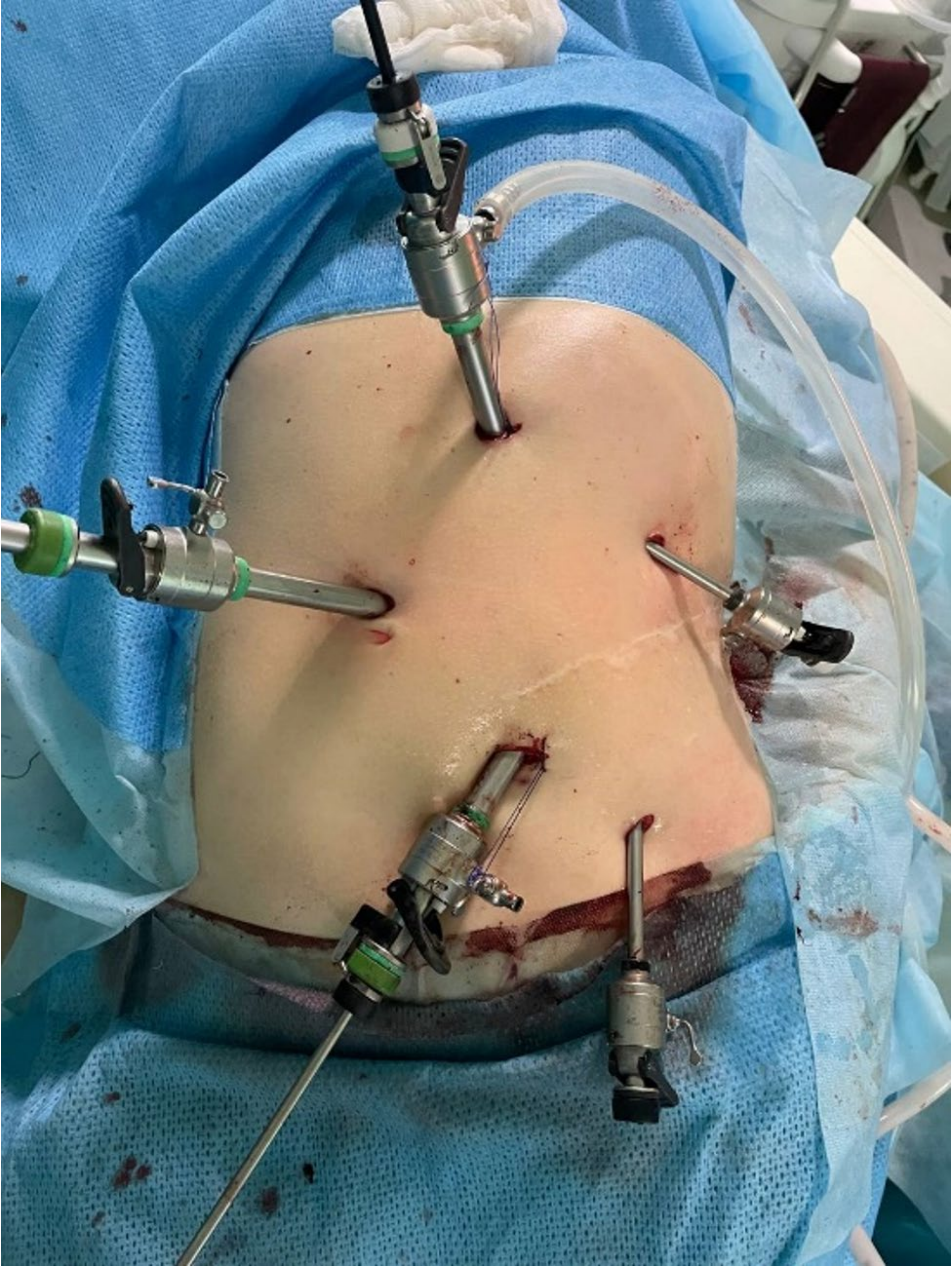
Intraoperative photography. Trocar placement

**Fig. 3:**
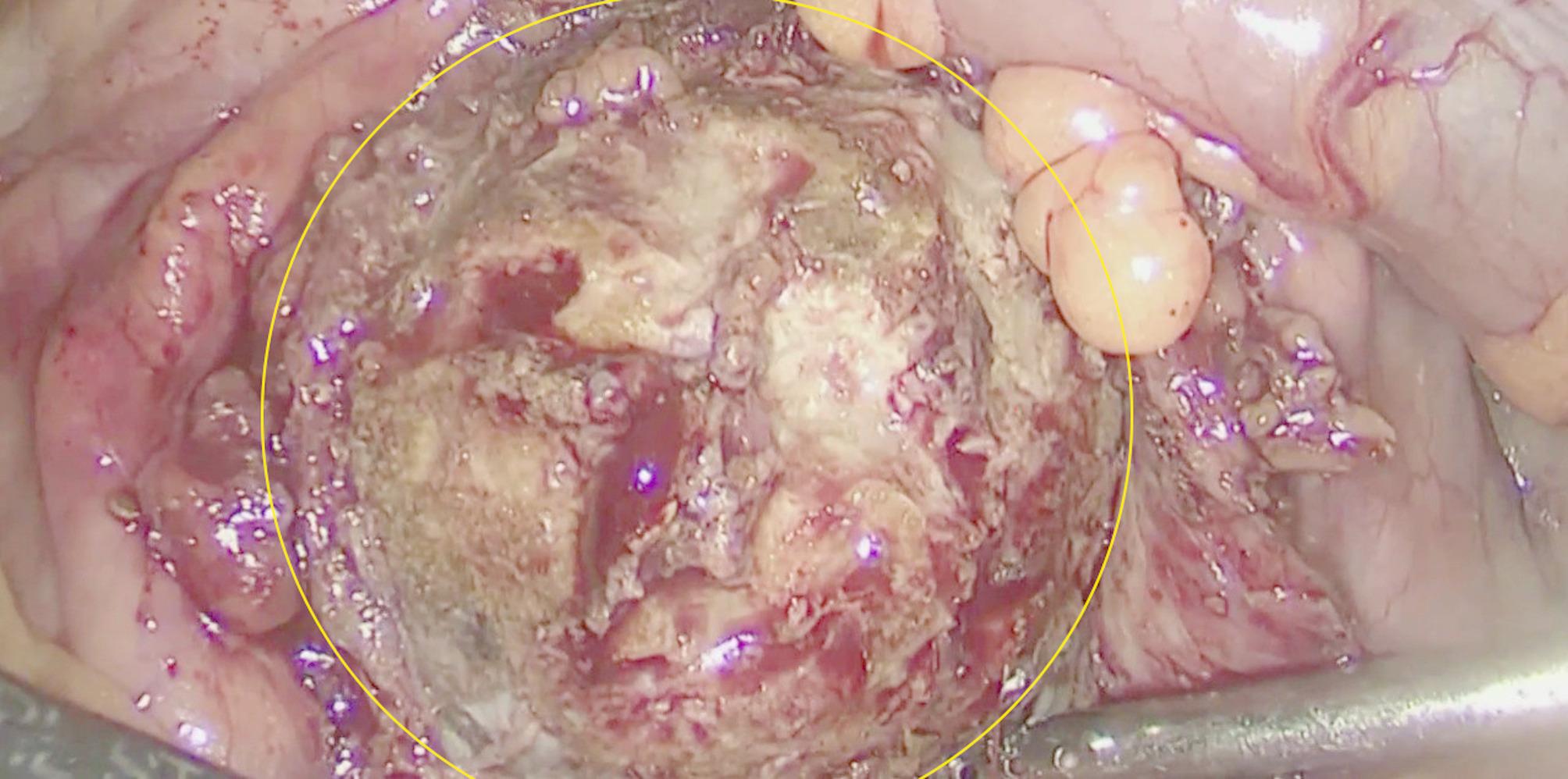
Intra-operative image of the stone. The area of the KS is circled in yellow

**Fig. 4:**
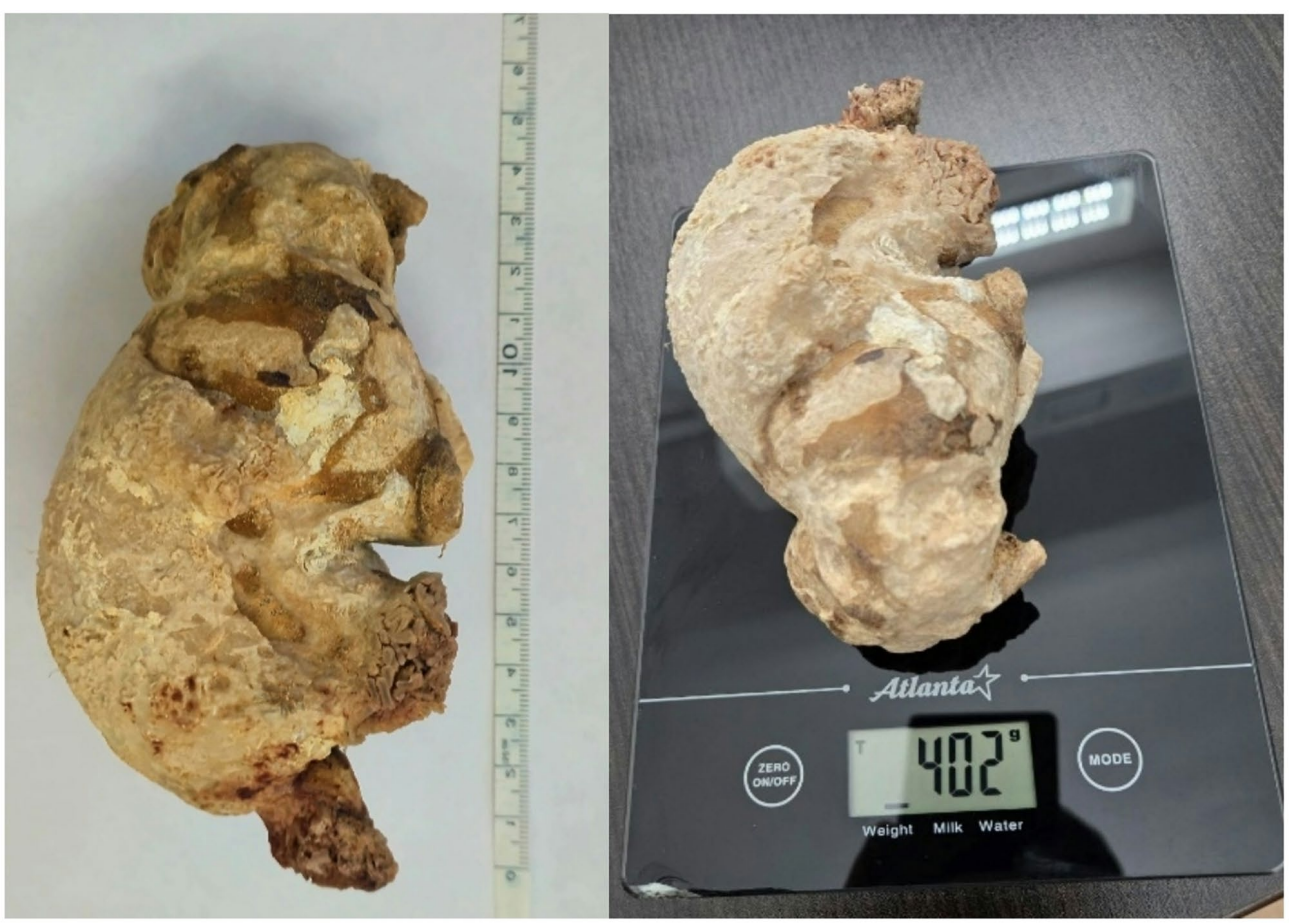
Stone size and weight

The surgical team consisted of an operating surgeon and two assistants. The Fig. 2 shows the trocar placement: five trocars were used to create a wider working space for this patient with a large stone. On examination, there were marked adhesions in the upper left abdomen, with the transverse colon in the splenic flexure adhering to the anterior abdominal wall over a distance of 7 cm. The pelvis of the left kidney could be seen, prolapsing through the visceral peritoneum. Access to the pelvis was achieved using a Harmonic Ace ultrasonic scalpel (Johnson&Johnson, USA) and a pyelotomy was performed. A yellow-brown KS was visualised in the pelvic cavity (Fig. 3) and removed. We restored the integrity of the renal pelvis with a continuous suture using V-Lock 3/0 thread (Medtronic-Covidien, Ireland) and then restored the integrity of the visceral peritoneum. The transperitoneal laparoscopic left pyelolithotomy was performed over 120 min with minimal blood loss. The stone (Fig. 4) was successfully extracted through a pararectal incision of approximately 8 cm in length.

**Fig. 5:**
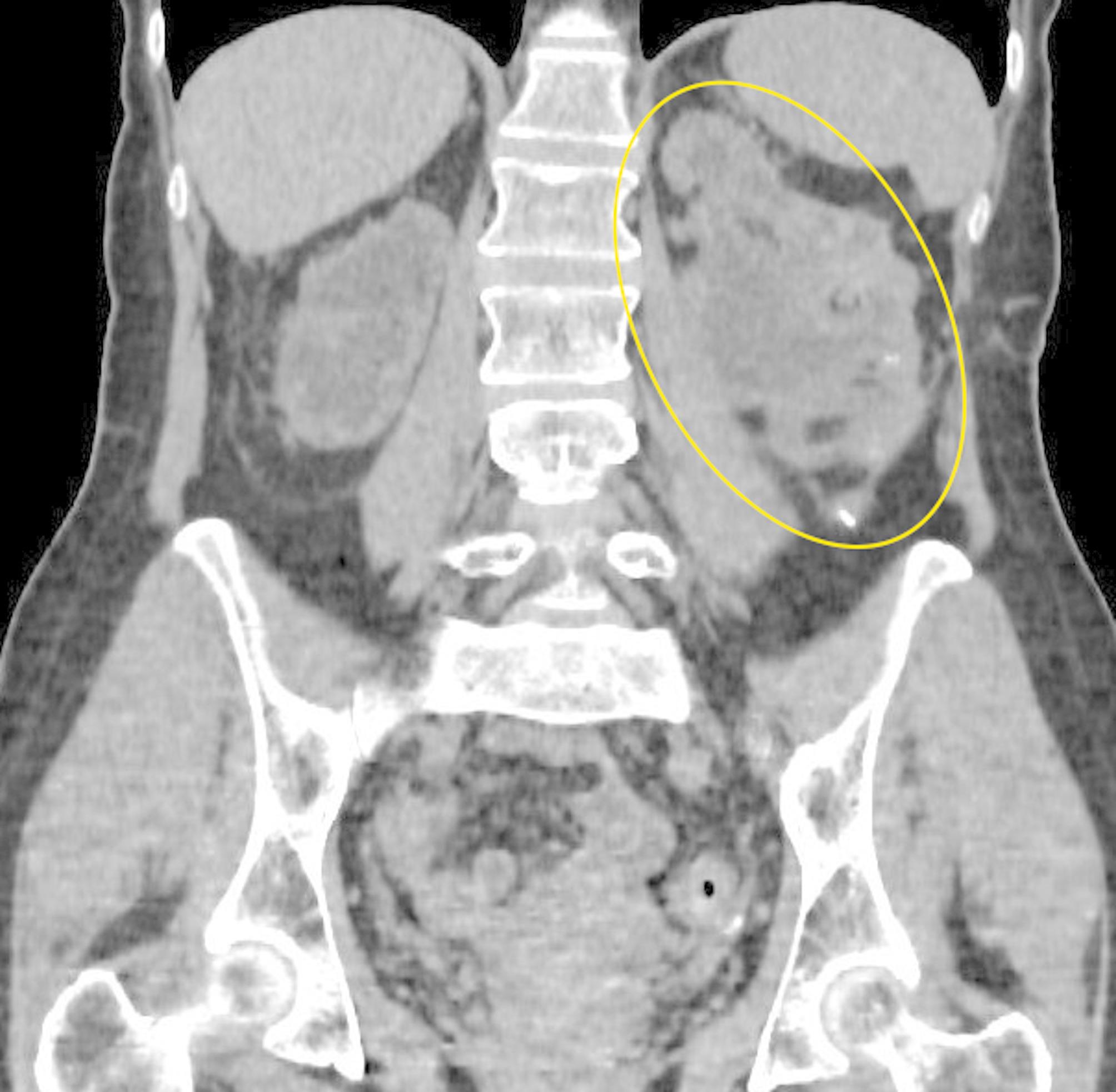
Computed tomography. The area of the left kidney is circled in yellow

We didn’t remove the calyceal KS because it didn’t not obstruct urine outflow and due to the risks of significant kidney injury. The patient’s post-operative recovery was uneventful and the drainage tube was removed on post-operative day 2. Eight months after surgery, creatinine levels and eGFR were 184 µmol/L and 35 ml/min, compared to 258 µmol/L and 23 ml/min prior to surgery. A follow-up CT scan performed 9 months after surgery demonstrates the success rate of the operation and is shown in Fig. 5.

## Discussion

The advent of improved diagnostic tools and minimally invasive treatment techniques has led to a notable decrease in the prevalence of KS greater than 100 g in modern urological practice. However, if left untreated, large KS can lead to significant complications, including kidney impairment and infection. While nephrolithiasis manifests more frequently in men, staghorn stones are reported less frequently in men than in women. Historically, 49–68% of staghorn stones were composed of struvite, however, recent studies indicate an increasing prevalence of calcium phosphate staghorn stones, thereby strengthening the association between staghorn stones and metabolic stone disease [4]. Furthermore, staghorn stones may manifest as mixed calculi, often composed of calcium carbonate apatite and struvite [5]. This phenomenon may be explained by an initial predisposition to calcium oxalate stone formation, which subsequently favours bacterial colonisation, triggering a cascade of events leading to secondary struvite deposition. Although there is no definitive explanation for the observed shift in stone composition, it is thought to be influenced by geographical, dietary and lifestyle factors.

Chronic nephrolithiasis is known to cause progressive kidney dysfunction. The pathological findings in renals with long-standing calculous disease include parenchymal atrophy, chronic pyelonephritis, xanthogranulomatous pyelonephritis, and, in rare cases, associated kidney malignancy. Li and Cheung et al. reported that 2% of patients with recurrent stones developed squamous cell carcinoma of the renal pelvis [6]. Chronic irritation of the urothelium in the upper urinary tract may result in proliferative changes, including squamous and glandular metaplasia. Giant KS typically result in kidney pelvic dilatation, kidney parenchymal atrophy and eventual loss of kidney function, often necessitating nephrectomy as the primary treatment modality.

In cases of salvageable renal parenchyma, kidney-preserving management approaches such as percutaneous nephrolithotomy (PCNL), laparoscopic nephrolithotomy, or open nephrolithotomy may be considered. A review of the literature indicates that due to irreversible loss of kidney function, frequent urinary tract infections and a chronic flank pain that cannot be managed by other treatment options most cases of giant KS are treated with nephrectomy. The largest documented KS treated without nephrectomy was a 1350-gram stone treated in Turkey in 2005. The largest KS removed by PCNL weighed 823.6 g and was reported in China in 2024 [7]. In another case, a 790-gram stone in a horseshoe kidney in India was treated with a combination of PCNL and open nephrolithotomy to preserve kidney function [8]. The largest stone successfully treated with PCNL alone was reported in Turkey in 2015, where 3 sessions of PCNL were performed over 3 months, requiring a total operative time of 460 min, 12 access tracts and one unit of blood transfusion [9].

The successful removal of a staghorn stone by laparoscopic pyelolithotomy demonstrates the significant advantages of this method in the treatment of large and complex stones. The primary benefits of this approach include the minimal trauma, reduced intraoperative blood loss, and decreased risk of intra-abdominal infection due to its use of miniature accesses. In addition, laparoscopy facilitates the preservation of the functional parenchyma of the renal, which is particularly important in patients with concomitant kidney failure. The short recovery time and early patient mobilization reduce the length of hospital stay and improve the patient’s quality of life. In contrast to open surgery and less invasive procedures, such as extracorporeal lithotripsy, the laparoscopic approach enables direct visualisation of anatomical structures, which is critical when dealing with massive stones that fill the pelvis and calyx. This approach enables the surgeon to meticulously remove the stone as a single unit, thereby mitigating the risk of fragmentation and residual fragments that frequently result in recurrence [10]. For large stones, where the efficacy of remote or transurethral lithotripsy is limited, laparoscopic pyelolithotomy is emerging as the method of choice, combining the radicality of open surgery with the advantages of minimally invasive technologies. Further improvement of the technique and accumulation of clinical experience will strengthen the position of this approach as the gold standard in the treatment of complex forms of urolithiasis [15]. 

The use of a retroperitoneoscopic approach for the removal of staghorn calculi remains a rare treatment option today. However, some authors have reported on the safety and effectiveness of this technique [11]. Due to the pronounced adhesive process after previous open retroperitoneal surgery, we used transperitoneal approach to reduce the trauma of the operation, the blood loss and the duration of the procedure.

Although PCNL remains the gold standard for the treatment of large and complex KSs due to its minimally invasive nature, common complications such as bleeding and infection remain a concern, particularly in cases involving extended procedures. Several studies suggest that the risk of bleeding and infectious complications increases with the duration of surgery [12]. Although percutaneous removal of such a large stone is feasible, it generally requires multiple access points. This carries a risk of kidney trauma due to injury to the renal parenchyma. For staghorn stones, laparoscopic nephrolithotomy has shown superior outcomes compared to PCNL [13]. The use of a retroperitoneoscopic approach for the removal of staghorn calculi remains a rare treatment option today. However, some authors have reported on the safety and effectiveness of this technique [11]. Due to the pronounced adhesive process after previous open retroperitoneal surgery, we used transperitoneal approach to reduce the trauma of the operation, the blood loss and the duration of the procedure. Based on these findings, laparoscopic pyelolithotomy was performed in the presented case with favourable results including minimal bleeding, absence of infectious complications and shorter hospital stay [13–15].

We found no articles in the available medical literature describing the use of laparoscopic approach for treating giant KS in case of reoperation.

## Limitations

The report has several limitations, including lack of generalizability and absence of control.

## Conclusions

Laparoscopic pyelolithotomy is a minimally invasive surgical technique employed for the removal of large and complex stones from the renal pelvis. The main advantages of the approach include a significant reduction in recovery time, minimal blood loss, reduced post-operative pain, and improved cosmetic results due to the minimally invasive nature of the procedure. In addition, laparoscopy is particularly relevant for patients with anatomical abnormalities, obesity, or recurrent stones, where alternative methods such as percutaneous nephrolithotomy (PCNL) or extracorporeal lithotripsy (ESWL) may be less effective or contraindicated. Laparoscopic pyelolithotomy remains an important tool in the urologist’s armamentarium, optimal for a certain category of patients. Further research should be focused on the optimization of indications for the procedure, the training of specialists, and the evaluation of long-term outcomes.

## Data Availability

We affirm that we had full access to all the study’s data. The datasets used and analyzed during the current study are available from the corresponding author on reasonable request.

## Data availability

We affirm that we had full access to all the study’s data. The datasets used and analyzed during the current study are available from the corresponding author on reasonable request.
